# The association between skin-suturing technique, prolonged healing and recurrence after Bascom's Cleft lift surgery

**DOI:** 10.1007/s10151-026-03377-7

**Published:** 2026-06-18

**Authors:** Marie Bjerg Larsen, Ida Kaad Faurschou, Susanne Haas

**Affiliations:** 1 U-COPE - University Clinic for Coloproctology and Endoscopy, Department of Surgery, Randers Regional Hospital, Randers, Denmark; 2 Aarhus University, Aarhus, Denmark; 3 Department of Clinical Medicine, Aarhus University Hospital, Aarhus, Denmark

**Keywords:** Bascom Cleft lift, Pilonidal disease, Pilonidal sinus, Recurrence, Wound healing, Suture techniques

## Abstract

**Background:**

The optimal treatment for complex Pilonidal Sinus Disease (PSD) is still debated. Despite great advances with the introduction of off-midline closure techniques, healing problems and recurrence remain challenges. We aimed to investigate whether choice of skin-suturing technique in Bascom’s Cleft lift (BCL) procedure influenced risk of prolonged healing or recurrence.

**Methods:**

We analysed the data of 540 unique patients who underwent BCL surgery at Randers Regional Hospital between 2016 and 2022. During this period, a technical revision of technique caused a shift from extracutaneous to intracutaneous closure creating two groups for comparison. Prolonged healing was defined as lack of complete epithelialization at the second post-surgical follow-up (maximum 14 weeks post-surgery).

**Results:**

There was no difference in time-to-healing among the suture groups with a 76% rate of primary healing in both groups. At 5 years post-surgery, the cumulative incidence proportion (CIP) of recurrence was 9.1% in the intracutaneous group and 5.7% in the extracutaneous group (*p* = 0.41). At 5 years post-surgery, the CIP of recurrence was 14.0% among patients with prolonged healing and 7.0% among patients with primary healing (*p* < 0.05).

**Conclusions:**

In our study population, choice of skin-suturing technique did not affect risk of prolonged healing nor recurrence after BCL surgery. However, patients experiencing prolonged healing after surgery had significantly higher risk of recurrence, emphasizing the need for increased efforts to prevent post-surgical wound dehiscence.

## Introduction

Pilonidal Sinus Disease (PSD) is a source for much morbidity both physically and mentally especially among young men, a population generally not associated with illness and disability [1, 2]. The incidence is approximately 57 per 100,000 person-years among men and 23 per 100,000 person-years among women as recently found in large a Danish cohort [3] and the peak age at incidence is between 15 and 24 years of age. New onset of disease after the age of 45 years is very rare [4].

PSD seem to occur when cut hairs become inserted into the *crena ani* owing to their barbed property, and the fragility of the skin in this part of the body [5]. This leads to a foreign body reaction with inflammation and cyst-like formation which may result in chronic inflammation with outlet or abscess formation in the absence of an outlet. Simple manifestations are treated with minimally invasive surgery, whereas extensive and complicated cases require excision and subsequent off-midline closure through mobilization of advancement flaps.

The invention of off-midline closure using advancement flaps radically improved the treatment of complicated PSD. Karydakis was the first to propose the hereafter-named ‘Karydakis flap’ [5]. This was followed by modified versions which includes the Bascom Cleft lift (BCL) [6]. Off-midline closure after surgical excision has been proven superior in comparison with open healing and midline closure on a wide range of points [7]. When suturing off-midline, you avoid suturing on top of the coccyx where the outwards forces are massive, and healing is difficult by default [8, 9]. However, even after off-midline closure surgery, complications related to healing and recurrence remain a source of frustration for surgeons and patients alike [10].

In the Central Denmark Region, Randers Regional Hospital serves as a referral centre for advanced PSD. At this centre, the BCL procedure is the standard treatment in cases not suited for a minimally invasive approach. Indications include extensive disease manifestation at the primary evaluation, recurrence of PSD after previous elective surgery or healing problems after previous surgery.

Since the establishment of a prospective database in 2016, all patients who underwent BCL surgery at Randers Regional Hospital up to 2022 have been systematically registered. During this period, two distinct skin-suturing approaches were employed. Until January 2018, extracutaneous closure was performed using single interrupted mattress sutures of monofilament, non-absorbable nylon (Dafilon^®^) removed 2 weeks post-surgery. After a technical revision of the procedure, skin closure was performed intracutaneously using a continuous (running) 3–0 Monosyn^®^ absorbable suture, eliminating the need for post-operative removal.

Little is known about how different skin-suturing techniques influence wound healing and the risk of recurrence following BCL surgery. John Bascom originally proposed to suture the skin with a subcuticular pullout of polypropylene, selected for its ease of removal (done 1 week after surgery) [6]. Our temporal variation offers a unique opportunity to compare the two patient groups and assess whether one suturing method is superior to the other.

## Materials and methods

### Study design

This is a retrospective analysis of prospectively collected data.

### Database and cohort

The study is based on a prospective database established in 2016 at Randers Regional Hospital using the Research Electronic Data Capture (REDCap) platform. All unique patients who underwent BCL surgery [6] and, for a smaller part, the Karydakis procedure [5] at the Department of Surgery at Randers Regional Hospital over a period from 2016 until the end of 2022 were consecutively registered. Follow-up was conducted in the clinic at 2- and 12–14-weeks post-surgery as well as regularly thereafter on the basis of necessity in cases of prolonged healing.

At our facility, we use autologous adipose tissue (AAT) for treatment of non-healing wounds – and during this period sometimes also as an add on to BCL surgeries. Patients who received BCL + ATT were excluded in this study as AATs are presumed to decrease the rate of healing problems following PSD surgery [11].

### Data collection

Data have been collected from when patients were initially referred for surgery and until healing. At the first consultation, when the indication for surgery was confirmed and the patient had agreed with the course of action, the following information was registered; civil registration number (CPR), gender, indication for surgery (extensive disease manifestation, recurrence or lack of healing after previous elective surgery), smoking habits, body mass index (BMI), significant comorbidity, American Society of Anesthesiologists Physical Status Classification System Score (ASA) and the number and nature of previous attempts at surgical management of the disease. On the day of surgery, the technical surgical details were added. These included, among others, the choice of suture used in skin-suturing and possible sub-procedures.

Information regarding healing and possible wound complications at the follow-up consultations 2- and 12–14-weeks post-surgery was registered on the day of the clinical visit, and additional visit were scheduled and registered, if necessary, until healing. On two separate occasions, all medical records were reviewed to register any additional treatment of PSD (recurrence). The latest audit was done in October 2025.

### Definitions and outcomes

For this study we, defined ‘recurrence’ as either reformation of pits or abscess formation indicating recurrent underlying disease, only in patients where complete healing had been previously verified by either a specially trained nurse or a doctor. This definition is in accordance with international literature [10, 12, 13], though there is controversy regarding the distinction between lack-of-heeling and recurrence [14].

The procedure was performed as day-case surgery. Hair removal was done via shaving peri-operatively, and patients were instructed in hair removal at home either by a nurse or relative 1–2 times weekly until complete healing, depending on hair density. Patients received antibiotics peri-operatively and for 3 days following surgery. Patients were informed to abstain from compromising activity e.g. biking, flexing of the hips and heavy lifting for the first 6 weeks post-surgery.

Suturing was grouped as either intracutaneous if the suturing was done subcuticularly using a resorbable (for the most part 3–0 Monosyn^®^) suture or extracutaneous if the suturing was done as transdermal mattress suturing using a non-resorbable (for the most part monofilament nylon suture, Dafilon^®^) suture.

Patients were grouped as either primary healers if they were healed with complete epithelialization by the second post-surgical follow-up (maximum 14 weeks post-surgery), or prolonged healers if they were not healed completely by then.

Follow-up was completed either on the day of registered recurrence, death or end of follow-up (1 October 2025), whichever came first.

### Statistical analysis

Of the 540 patients in the cohort, complete data were available for 437. Analyses were conducted using all available data for each variable; patients with missing information for a given element were included in analyses for which data were present and excluded only from analyses requiring the missing variable. As a result, sample sizes varied across analyses.

Data was summarized and presented as medians with either inter quartile range (IQR) or range, dependent on a qualitative assessment of the most informative presentation.

Cumulative incidence proportion (CIP) plots were generated to describe the time-to-event probabilities for recurrence and prolonged healing across subgroups.

Results from a Cox regression linear model were summarized in a forest plot showing hazard ratios (HRs) with 95% confidence intervals (CIs) for covariates.

A *p*-value of < 0.05 was considered significant.

Analysis and illustrations were conducted using RStudio version 2023.12.1.

## Results

Overall patient demographics and surgery characteristics are shown in Table 1.

**Table 1:** Demographics and surgery characteristics of patients undergoing BCL surgery at Randers Regional Hospital between 2016 and 2022

Characteristic	*n* = 540^1^
Gender Female Male	83 (15.4%) 457 (84.6%)
Age	23 (19, 28)
Age at debut of disease *Missing*	20 (17, 24) *49*
ASA^2^ 1 2 3 *Missing*	405 (76.7%) 112 (21.2%) 11 (2.1%) *12*
BMI^3^ *Missing*	26.3 (23.7, 29.8) *34*
Tobacco Active smoker Former smoker (> 6 weeks since smoking) Never smoker *Missing*	46 (8.7%) 160 (30.4%) 321 (60.9%) *13*
Indication for surgery Extensive manifestation (no previous surgery) Healing problems after previous elective surgery Recurrence after previous elective surgery	189 (35.0%) 177 (32.8%) 174 (32.2%)
Skin suture Intracutaneous Extracutaneous *Missing*	329 (70.0%) 141 (30.0%) *70*
Time-to-healing Primary healing Prolonged healing *Missing*	403 (75.8%) 129 (24.3%) *8*
Follow-up, months	69 (50, 92)

^1^*n* (%); Median (IQR)

^2^ASA: American Society of Anaesthesiologists Physical Status Classification System

^3^BMI, Body Mass Index

Patient groups on the basis of skin suturing were comparable on all relevant parameters apart from follow-up time. Patients who were sutured extracutaneously had a median follow-up of 100 months (IQR = 94, 105), whereas patients who were sutured intracutaneously had a median follow-up of 59 months (IQR = 44, 76).

**Fig. 1:**
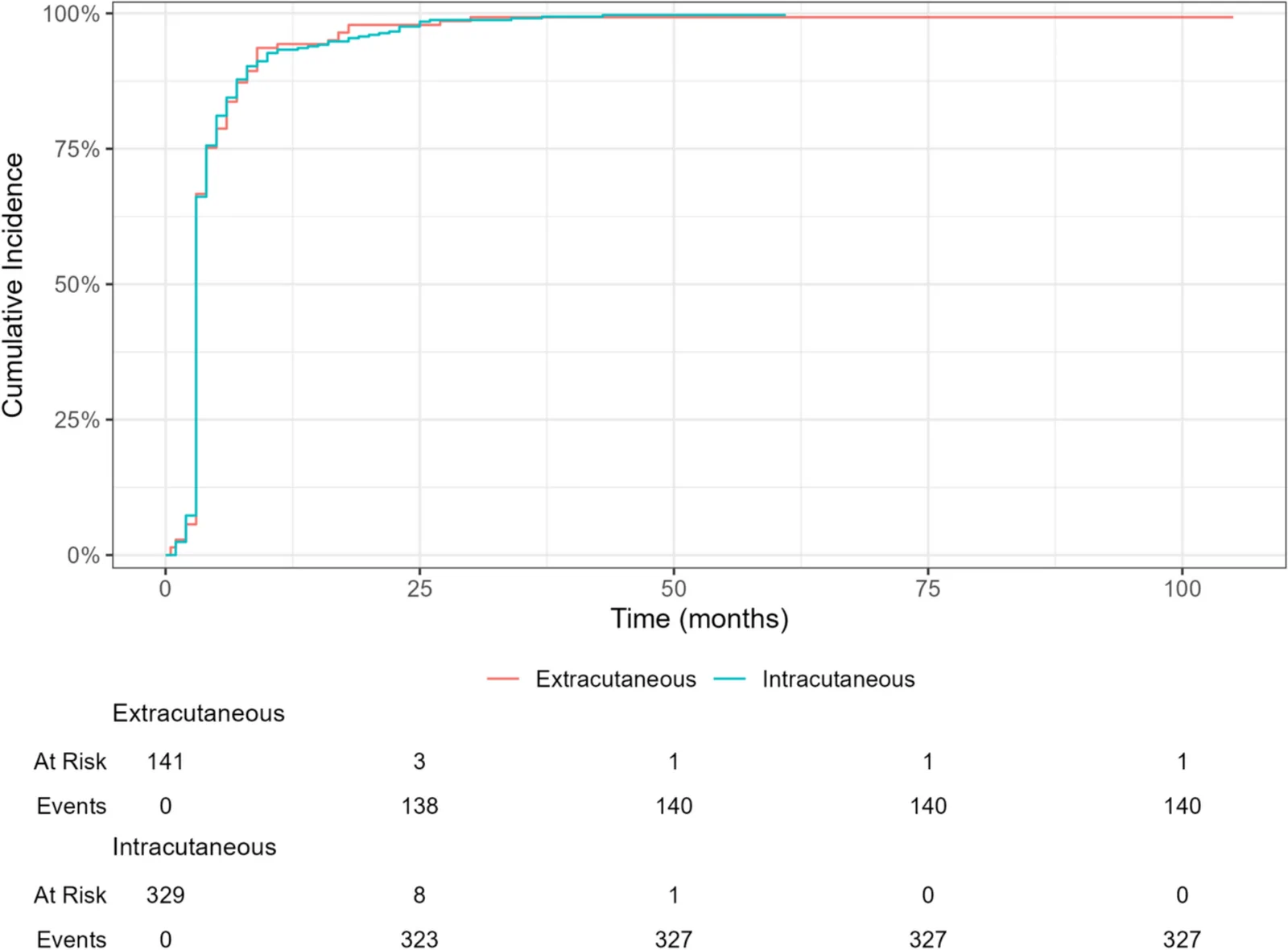
CIP-plot, cumulative incidence proportion of healing among those sutured extracutaneously (red curve) and those sutured intracutaneously (blue curve) as a function of time in months

We found a similar cumulative incidence proportion of healing between the two groups with a median time to heal of 3 months (range = 0.5–30.0) for the extracutaneous group and 3 months (range = 1.0–43.0) for the intracutaneous group (*p* > 0.9) (Fig. 1). In both groups, 76% of patients were primary healers. At 25 months post-surgery, healing was obtained for 98.1% of all patients.

**Fig. 2:**
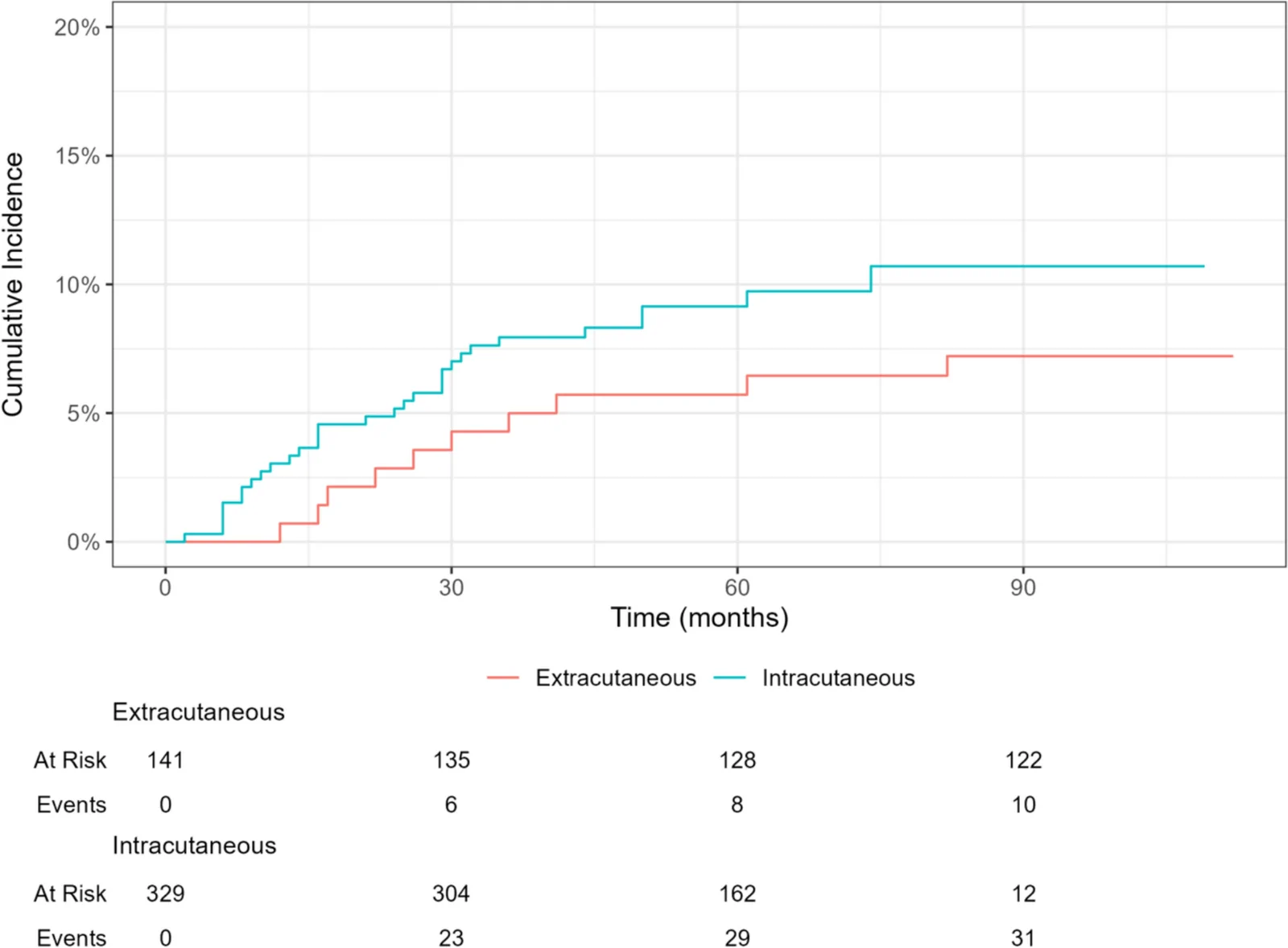
CIP-plot, cumulative incidence proportion of recurrence among those sutured extracutaneously (red curve) and those sutured intracutaneously (blue curve) as a function of time in months

The CIP of recurrence over time differed slightly among the extracutaneous and intracutaneous suture groups (Fig. 2). Since the groups differed in follow-up duration (median (IQR) 100 months (94, 105) versus 59 months (44, 76)), comparisons were made using the CIP at 5 years (60 months) post-surgery. At 5 years, the CIP of recurrence was 5.7% (95% CI: 2.7–10.0%) in the extracutaneous group and 9.1% (95% CI: 6.3–13.0%) in the intracutaneous group; however, the higher hazard of recurrence in the intracutaneous group was statistically non-significant (*p* = 0.41) (Fig. 4). Median time to recurrence in months was respectively 26 [range = 4–82] in the extracutaneous group and 21 [range = 2–74] in the intracutaneous group (*p* = 0.3).

**Fig. 3:**
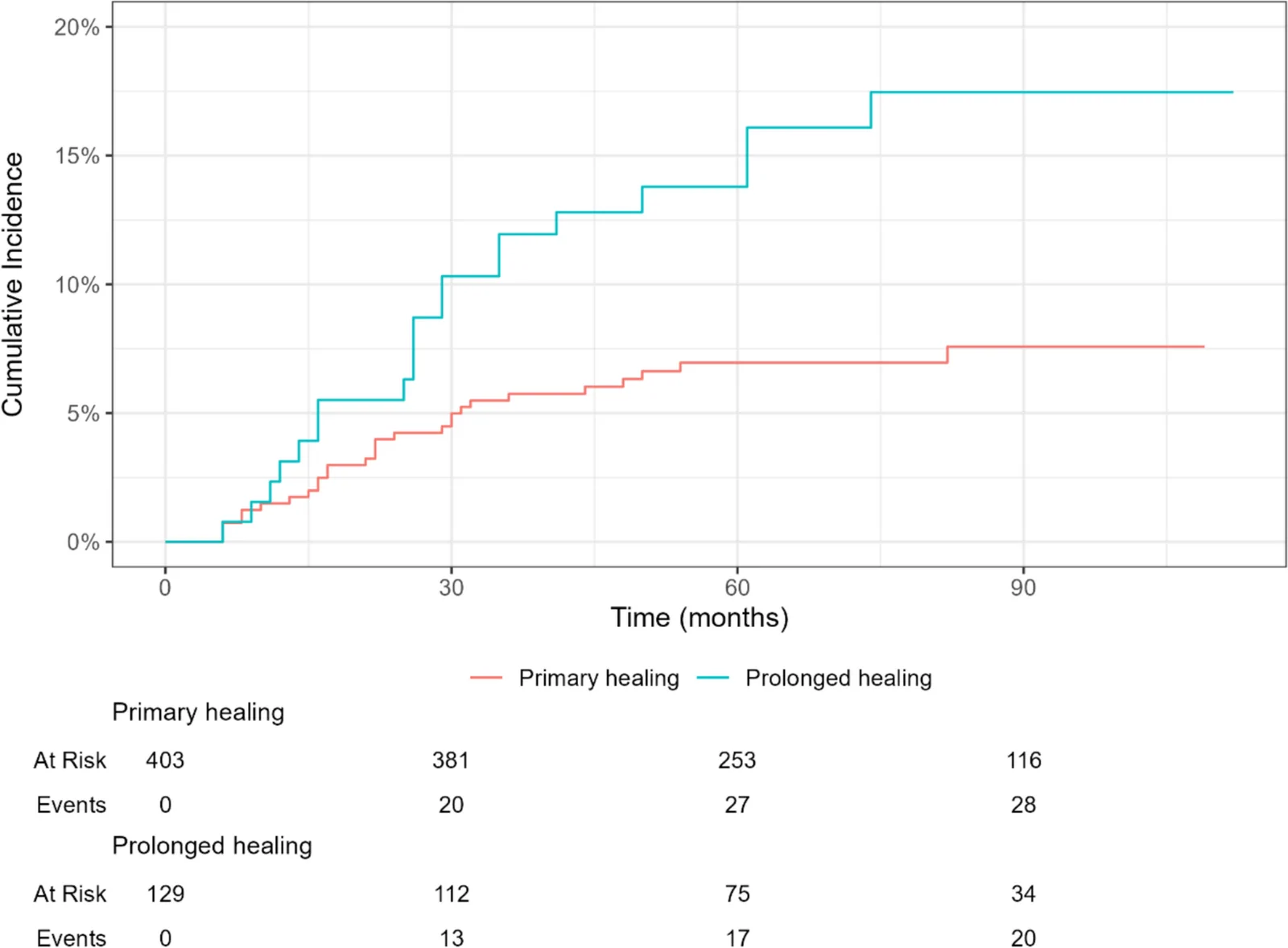
CIP-plot, cumulative incidence proportion of recurrence among patients with primary healing (red curve) and prolonged healing (blue curve) as a function of time in months

**Fig. 4:**
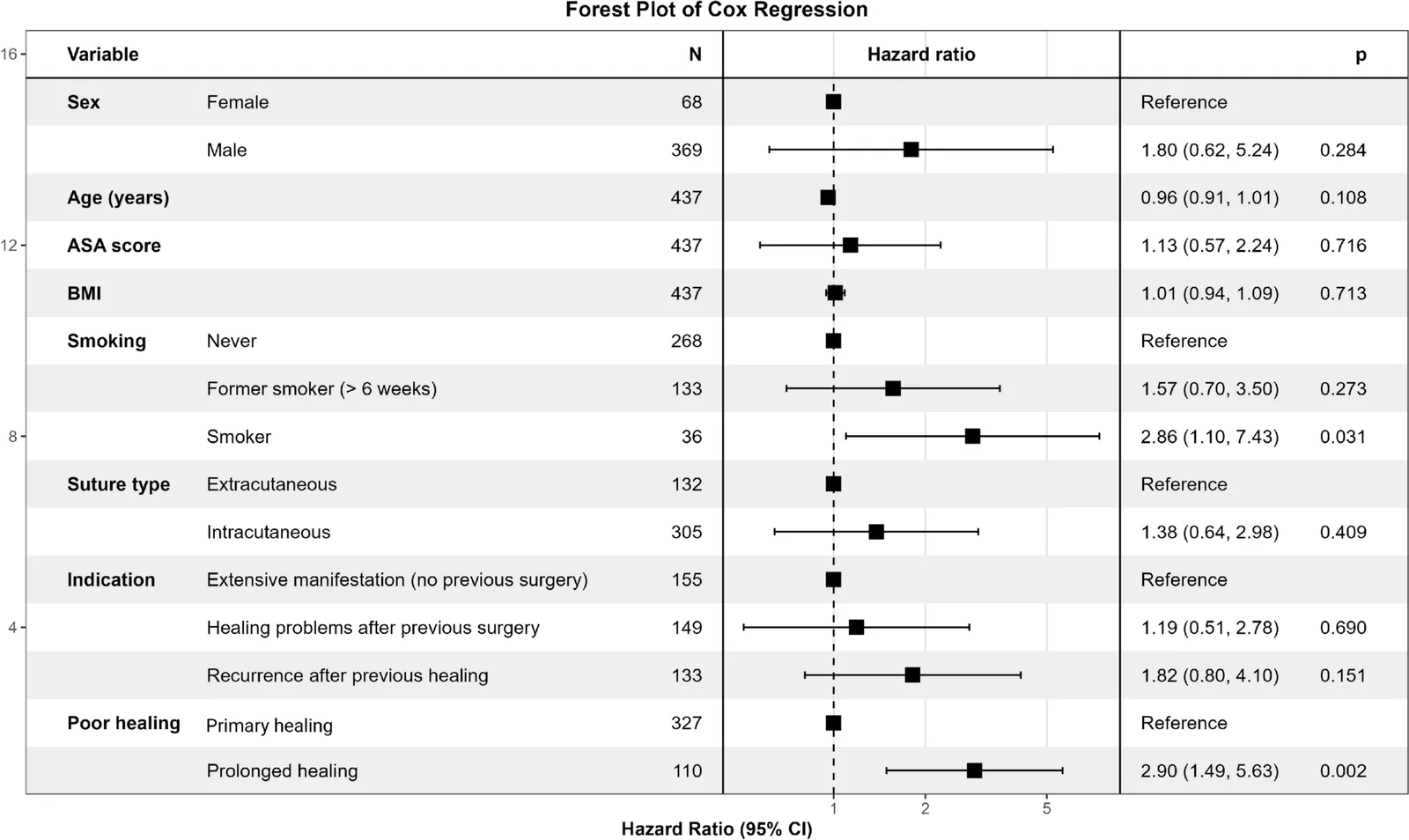
Forest plot of a Cox regression linear model examining variables associated with recurrence. Only 437 patients are included in this analysis, since the remaining 103 patients each have missing data on either one or more variables of the model. The forest plot presents hazard ratios for multiple variables associated with recurrence, each with corresponding 95% confidence intervals. *p*-values are calculated using the Wald-test

The CIP of recurrence differed between patients with primary healing and those with prolonged healing. At 5 years after surgery, the CIP of recurrence was 7.0% (95% CI: 4.7–9.8%) in the primary healing group and 14.0% (95% CI: 8.4–21.0%)(Fig. 3) in the prolonged healing group. Compared with primary healing, prolonged healing was associated with a significantly higher hazard of recurrence (Fig. 4). The median time to recurrence in months was 22 [range = 6–82] for the primary healing group and 26 [range = 4–74] for the prolonged healing group (*p* = 0.6).

Active smoking was significantly associated with an increased hazard of recurrence compared with never smoking (Fig. 4). No surgical indication was significantly associated with recurrence; however, patients undergoing BCL owing to recurrence after previous healing had a higher, but non-significant, hazard of recurrence compared with those treated for extensive primary manifestation (Fig. 4).

## Discussion

We did not find that method of skin closure (extracutaneous versus intracutaneous) affected the primary healing rate, nor did we find it to significantly affect recurrence; however, a trend of a higher risk of recurrence among those of our patients who were sutured intracutaneously was found. More importantly, we found patients who experienced prolonged healing were at a significantly higher risk of recurrence than those who healed primarily. Active smoking was also significantly associated with an increased risk of recurrence compared with never smoking.

### Skin-suturing method and risk of recurrence

Choice of skin-suturing method at Randers Regional Hospital has shifted during the inclusion period from primarily extracutaneous suturing until 2018 to primarily intracutaneous suturing from 2018 till 2022. This explains the substantial difference in follow-up duration between the groups. Our data suggests that the observed trend (however statistically non-significant) towards a difference in rate of recurrence is not the result of a propensity among one method of skin-suturing to result in poor and prolonged healing.

The efficacy of suturing intracutaneously rather than extracutaneously upon closing the wound in BCL surgery has not been formally investigated to our knowledge, but other modifications to the original instruction by John Bascom [6] have been suggested with positive results [15, 16]. A 2013 study by Milone et al. compared intradermal absorbable suturing with interrupted suturing in excisional PSD surgery with primary closure [17]. Follow-up was concluded at 6 months, so no long-term data on recurrence is available. During healing, infection rates were similar among the two groups and patients in the intradermal group were generally more satisfied with the result of their surgery. The findings regarding comparable healing rates between suturing groups align with our own.

A large Cochrane review from 2016 of studies which compared subcuticular suturing with transdermal suturing in non-obstetric surgeries found that overall patient satisfaction was moderately higher among the subcuticular group, and wound infection rates were similar [18]. Patient satisfaction was rated on the basis of factors such as cosmesis, hygiene problems, tension on wounds and overall well-being. While this review did not focus on BCL surgery, it highlights the point that patient satisfaction depends on matters besides time-to-healing and low rates of recurrence.

Since we have found no causative explanation for the non-significant difference in rate of recurrence, whether to suture skin intracutaneously or extracutaneously should depend on anatomical and functional considerations as well as the surgeon’s preference and experience, until further investigation confirms a significant advantage to either method.

### Prolonged healing and risk of recurrence

Prolonged healing and healing problems in general after BCL surgery is a common issue [10, 19–21]. Our definition of prolonged healing as lack of complete epithelialization at the second post-surgical follow-up at maximum 14 weeks post-surgery is not derived from international literature. Rather, it is the natural cut-off for primary healing, since the standard follow-up regimen at our centre is concluded at 12–14 weeks post-surgery. Therefore, the proportion of ‘prolonged healing’ in our cohort cannot be directly compared with the corresponding proportions reported in other studies. A 2024 study by Brown et al. compared the outcomes of patients across 31 centres, who had undergone either major excisional surgery (BCL, Karydakis etc.) or minimally invasive surgery (pit-picking, sinus curettage with glue etc.) for PSD. Complete healing post-surgery was only achieved in 75% of cases regardless of group. During the study period of 300 days, most of these patients had healed by day 180 (6 months). Brown et al. do not report on recurrence, as it is not within the scope of their study, but estimate the incidence of true recurrence to be uncommon [21].

Although the operative techniques differ from BCL, other studies have reported on the significance of healing problems in the rate of recurrence after PSD surgery. In 2002, Søndenaa et al. reported on a combined recurrence rate from two different studies which examined the effect of antibiotic prophylaxis in PSD surgery. The operative technique is described as excision with primary closure, uncertain location. The group found a significantly increased risk of recurrence in patients who had experienced primary wound problems, regardless of infection [22].

A 2015 study by Doll et al. compared the recurrence rates after PSD surgery, primary midline closure, between those patients who had post-operative wound ruptures with patients who had uneventful healing. No difference in recurrence rate was seen if the ruptures were treated secondarily open [23].

Recurrence rates after BCL surgery differ tremendously in international literature, but as recently concluded in a large meta-analysis recurrence rates impressively depend on follow-up time [8]. A review and meta-analysis by Milone et al. concerning studies reporting at least 5 years follow-up after surgical management of PSD, found a general recurrence rate after off-midline flap-surgery of 10% [24], which is in accordance with our overall findings.

In a series of 700 patients undergoing BCL by one single surgeon, S. Immerman, the rate of success after the initial surgery was 96.1% (no early or late failure, need for revision or recurrence of disease) [16]. Median follow-up was 6 months; however, this impressive rate of success underlines the importance of expertise in handling complex cases of PSD. Our findings call for an increased focus on bringing down the rates of prolonged healing following complex PSD surgery.

### Smoking and risk of recurrence

In our cohort, active smokers at the time of surgery had nearly a threefold higher risk of recurrence compared with never smokers. A 2013 study by Sievert et al. found no relation between smoking and wound complications or risk of recurrence after PSD surgery. A key difference however; the surgery in question was either primary midline closure or primary open treatment, and recurrence rates after 20 years of follow-up ranged from 28–40% in all groups [25].

In 2020, the World Health Organisation (WHO) released a large review on surgical outcomes in general in smokers versus non-smokers with the overwhelming conclusion that smoking increases the risk of adverse post-operative outcomes [26]. The mechanisms through which smoking adversely affects surgical site healing are thought to concern increased peripheral tissue hypoxia, decreased inflammatory responses, delayed proliferative healing responses and reduced collagen synthesis [26].

As we have identified prolonged healing as a risk factor significantly associated with recurrence of PSD after BCL surgery, prolonged healing may constitute an intermediary link between active smoking and an increased risk of recurrence.

All patients should be encouraged to cease smoking prior to surgery. Our data on the patients’ smoking status was disclosed by the patients, and this leaves room for under-reporting. Current guidelines at our centre dictate that patients must cease smoking at least 8 weeks prior to BCL surgery and remain abstinent until 6 weeks post-surgery or until healing.

### Clinical perspectives

To our knowledge, ours is the first study to examine the correlation between choice of suture method, prolonged healing and risk of recurrence after BCL surgery. Our finding that prolonged healing significantly increases the risk of recurrence of disease, narrows in on the need for further investigation into what factors, which may be within technical control, can be adjusted to aid patients in primary uncomplicated healing.

Our mean follow-up time was 69 months with large variations among groups. As evident from our results, recurrences occur even after 69 months post-surgery, even though the incidences decrease hereafter. Future studies into similar aspects of the treatment of PSD with longer follow-up are warranted to validate and put our findings into perspective.

### Limitations

Though we were privileged to examine a large cohort, and data have been, primarily, gathered prospectively, this study has certain limitations. Patients who received sub-procedure AAT transplants were excluded from our cohort. The practice of AAT transplants has been gradually implemented from 2019 in the department of surgery at Randers Regional Hospital as a treatment offer for patients with non-healing wounds > 2 months after PSD surgery or as a sub-procedure during the Basom Cleft Lift surgery, if patients are presumed to have a high risk of poor healing and subsequent complications. Indications include peri-anal disease manifestation, high BMI or multiple recurrences [11]. Given the indication for the sub-procedure, we are aware that we will have excluded some patients who would statistically be at risk of a poor outcome of the BCL surgery regarding both prolonged healing and risk of recurrence, and by excluding the group, we run the risk of underestimating both parameters.

In addition, given the median age of 23 at the time of surgery, the cohort represents a young population, many of whom are highly mobile in their living arrangements. Since our search for recurrence of disease has been limited to the hospitals of the Central Denmark Region, we are at risk of missing recurrent disease, which may have occurred and been treated at a hospital in another Danish region or outside of the country.

A total of 103 patients had partially missing data and were thus excluded from one or more analyses. Missing data was relatively evenly distributed among suture and healing groups. All data was registered by healthcare professionals either prospectively or retrospectively through journal audits, hereby markedly diminishing the risk of selection bias.

While these limitations should be acknowledged, we believe our findings remain reliable and are unlikely to be substantially impacted.

## Conclusions

Our finding suggest that extracutaneous closure during BCL surgery may reduce risk of recurrence; however, this trend does not appear to be mediated by an increased likelihood of prolonged healing among patients who were sutured intracutaneously. We found that prolonged healing itself was significantly associated with an elevated risk of recurrence compared with primary healing. These findings highlight the need for further investigation into factors within technical control, that can be adjusted to aid patients in primary, uncomplicated healing.

## Data Availability

The data that support the findings of this study are not publicly available owing to privacy or ethical restrictions. No datasets were generated or analysed during the current study.
